# Feedback-Controlled Release of Alendronate from Composite Microparticles

**DOI:** 10.3390/jfb11030046

**Published:** 2020-07-01

**Authors:** Sofia S. H. Matrali, Anita K. Ghag

**Affiliations:** School of Chemical Engineering, University of Birmingham, Birmingham B15 2TT, UK; sophy_sophie@yahoo.com

**Keywords:** bone, bisphosphonate, biodegradable, drug delivery, controlled release, composite

## Abstract

Extended bone fractures or fractures coexisting with bone disorders can lead to non-unions where surgical intervention is required. Composite drug delivery systems are being used increasingly more in order to treat such defects locally. Alendronate (ALD), a bisphosphonate extensively used in clinical practice to treat conditions, such as osteoporosis, has been shown to assist bone fracture healing through its antiresorptive capacity. This study reports the development of a polymeric composite system for the in situ delivery of ALD, which possesses enhanced encapsulation efficiency (EE%) and demonstrates controlled release over a 70-day period. ALD and calcium phosphate (CaP) were incorporated within poly (lactic-co-glycolic acid) (PLGA) microspheres, giving rise to a 70% increase in EE% compared to a control system. Finally, a preliminary toxicological evaluation demonstrated a positive effect of the system on pre-osteoblastic cells over 72 h.

## 1. Introduction

Bone performs a variety of tasks critical to human physiology [1]. Apart from its most commonly acknowledged role as the body’s structural component, bone tissue also performs other functions, such as the protection and support of other internal organs, production of blood cells, and storage of mineral salts [2]. Bone tissue possesses some unique properties, such as self-remodeling and regeneration via structural and formational tissue restoration [3,4,5,6]. In complex cases, damaged or diseased tissue is unable to regenerate, and, in such cases, surgical intervention is required.

In the past, non-healing fractures were addressed surgically using rigid fixation devices, which lack biodegradability but also restrict healing [7]. Research then shifted to the development of bone grafts, namely autografts or allografts [1,2,5]. However, both types of graft are associated with limited availability, prolonged recovery, donor-site morbidity, and disease transmittance. There is a clinical need to develop a synthetic alternative [8,9].

Tissue engineering is a multidisciplinary field based on materials science and molecular biology [10]. The field focuses on the development of biological substitutes to replace damaged or diseased tissue or to assist new tissue formation [11]. Within bone tissue engineering, such devices focus on the activation of natural repair mechanisms [5,12]. A vast range of materials have been studied for the formulation of such devices [2,5,7,13], including natural or synthetic polymers and ceramics, either individually or in combination with bioactive compounds (growth factors, proteins, etc.) and/or cellular components (mesenchymal stem cells). Research has shown that amongst the most successful are composite-based devices, which incorporate hydrophilic polymers and inorganic mineral like hydroxyapatite (HA). It is suggested that the incorporation of drugs, such as bisphosphonates (BPs), could further increase the integration of devices within the host environment and subsequently reduce surgical recovery times [6,14].

Alendronate (ALD) is a type of BP and is clinically used to treat bone disorders, such as osteoporosis [15,16]. Recently, it has been suggested that the introduction of ALD in osteoporotic bone fractures could improve fracture healing [17]. Alendronate has been found to bind and thus block “… an enzyme in the 3-hydroxy-2-methylglutaryl-CoA (HMG-CoA) reductase pathway (i.e., the mevalonate pathway), thus blocking the prenylation of small GTPases …” as described by Kyllönen [17]. GTPases are hydrolytic enzymes controlling the conversion of guanosine triphosphate (GTP) to guanosine diphosphate (GDP) [18,19]. Ultimately, this has a detrimental effect on the function and viability of osteoclasts, leading to a net increase in tissue.

Poly (lactic-co-glycolic acid) (PLGA) is a synthetic copolymer of polylactic acid (PLA) and polyglycolic acid (PGA), which has been adopted in the production of various therapeutic devices, including tissue grafts, surgical sutures, bone tissue engineering scaffolds, and drug carrier systems, due to its excellent biocompatibility and ability to tune degradation [1,5]; the degradation rate can be tailored based on the ratio of lactic to glycolic acid. Both PLA and PGA degrade via non enzymatic hydrolysis, which is characterized by auto-catalysis due to the acidic nature of the degradation products (carboxylic acids), and this constitutes a common feature amongst a-polyesters [20]. Co-polymerization of lactic and glycolic acids is used to improve the degradation pattern. Their copolymers, PLGA polymers, offer the possibility of controlling the degradation rate of the product by adjusting the glycolic to lactic acid ratio, consequently controlling the release profile of bioactive molecules transported in relevant carriers [21]. Degradation progresses through bulk erosion of ester bonds and it is also affected by the architecture of the devices as well as the entrapped moieties. Unfortunately, the absence of a linear correlation between the copolymer’s composition and its degradation profile has been shown; for example, when the ratio is 50% PLGA, the system degrades faster than both PLA and PGA. PLGA is U.S. Food and Drug Administration approved for use in humans and has been prepared into several different formulations, including scaffolds, hydrogels, nanoparticles, microparticles, and sponges. Consequently, PLGA is an attractive choice for bone regeneration [5].

Researchers have attempted to deliver ALD to local sites; however, achieving controlled release can be challenging due to the high solubility of the drug. In this study, we aimed to create a composite system for the local delivery of ALD to fracture sites via controlled release. This was achieved through the development of PLGA and calcium phosphate (CaP) microparticles, which were loaded with the active drug. CaP was selected in order to prolong the release of ALD, which is currently challenging, through the formation of a mineral shell, which is hypothesized to decrease the degradation rate of the system. This system would allow for the delivery of a drug to fracture sites, thus leading to a net increase in bone formation. Although out of the scope of the current study, the composite system could be incorporated within a hydrogel system to form an injectable material that could be delivered to the site of injury.

## 2. Materials and Methods

### 2.1. Materials

Alendronate sodium salt (purity ≥ 95%) was purchased from Cayman Chemical (Ann Arbor, MI, USA). Poly (lactic-co-glycolic acid) 50% was purchased from Lakeshore Biomaterials (Birmingham, AL, USA). Dichloromethane (DCM) was purchased from Fisher Chemicals (Hampton, NH, USA). Nitric acid (HNO_3_) and poly (vinyl alcohol) (PVA) (molecular weight 31,000–50,000, 98–99% hydrolyzed) were purchased from Acros Organics (Fair Lawn, NJ, USA). All other chemicals and reagents were purchased from Sigma-Aldrich (Gillingham, UK).

### 2.2. Methods

#### 2.2.1. CaP Particle Formation

CaP particles were prepared following a wet precipitation method as described by Mobasherpour [22]. Briefly, a 0.29 M aqueous solution of (NH_4_)_2_HPO_4_ was added dropwise to 0.24 M aqueous solution of Ca(NO_3_)_2_.4H_2_O at a volume ratio of 1.4 Ca(NO_3_)_2_.4H_2_O to (NH_4_)_2_HPO_4_. Synthesis was performed under stirring under atmospheric conditions. Mixing was performed using a homogenizer (IKA^®^ T25 digital, ULTRA TURRAX, Berlin, Germany*)* and the solution was maintained at pH 10. The precipitate was washed with distilled water and collected by centrifugation at 3000 rpm in 15-min cycles. Homogenization was performed at different speeds and durations and the size distribution was examined at various time points under atmospheric conditions.

##### Physicochemical Characterization

The size distribution of the CaP particles was performed using a Malvern Mastersizer 2000 (Malvern Panalytical Ltd., Malvern, UK) via light scattering and the surface charge was evaluated using a Malvern Zeta-sizer (Nano-ZS) using the Smoluchowski model. Prior to measurement, samples underwent sonication for one hour to break down aggregates.

The chemical composition of the CaP particles was determined via Fourier transform infrared spectroscopy (FT-IR) and X-ray fluorescence (XRF). For FT-IR analysis, sample pellets were formed into mechanical mixtures of 1% mineral particle powder in potassium bromide (KBr). CaP particles were thoroughly dried before analysis. For XRF analysis (50 kV, 300 μA, 25 μm spot size, 20 mbar vacuum), samples were dried and pressed into pellets.

#### 2.2.2. ALD-Loaded Microspheres

The microspheres (Msps) were prepared using a water-in-oil-in-water (w/o/w) double emulsion-solvent evaporation method [23]. Briefly, 2.25 mL of PLGA 50% solution 2% w/v in dichloromethane (DCM) were emulsified with 0.45 mL of 10% w/v PVA aqueous solution. For the preparation of ALD-loaded microspheres, ALD was initially dissolved in the inner aqueous phase. For the preparation of CaP-ALD-loaded particles, ALD was mixed with CaP particles’ aqueous dispersion and allowed to bind for 24 h, and then mixed with 10% w/v PVA solution. The emulsification was accomplished using a homogenizer (IKA^®^ T25 digital, ULTRA TURRAX) at 8000 rpm for 30 s. The primary emulsion was further emulsified using a magnetic stirrer with 42.2 mL of 10% w/v PVA aqueous solution for 4 h until the organic phase fully evaporated. Microspheres were washed with water to remove residual PVA and non-encapsulated drug, and collected using centrifugation (JOUAN C422 centrifuge, Thermo Fisher Scientific) at 4000 rpm using 30-min cycles. The process was repeated until the supernatant was clear and transparent. Finally, the microspheres were dried under vacuum at room temperature for 24–48 h (Edwards High Vacuum, V&IP, Sussex, UK).

##### Microspheres’ Physicochemical Characterization and Drug Encapsulation

Spectrophotometric evaluation (CE 7500 Double Beam UV/Visible Spectrophotometer) of the polymeric microspheres was carried out to calculate the ALD content. The method used was based on the formation of an ALD-Cu(II) chromophoric complex absorbing at 240 nm according to the work described by Ostovic [24,25,26].

Drug encapsulation in solid microspheres: 2 mg of particles were dispersed in 0.5 mL of DCM. Then, 2 mL of nitric acid (HNO_3_) 1.5 mM were added and the mixture was stirred at 160 rpm for 20 min. The aqueous phase was filtered, and 1 mL was diluted with 1 mL of 1.5 mM HNO_3_ and 2 mL of Cu^+2^ 5 mM solution in 1.5 mM HNO_3_. The mixture was vortexed for 30 s and left in the dark for 20 min. The sample’s absorbance was measured at 240 nm and used for calculating the ALD concentration.

##### Size Distribution and Morphology

The size distribution of the fabricated microspheres was determined using optical microscopy (ZEISS optical microscope) and a particle sizer (Malvern Mastersizer 2000). Particle morphology was further evaluated using scanning electron microscopy (SEM) (JEOL JSM-6060LV). For SEM, samples were dried and mounted on aluminum stubs and then subjected to platinum sputtering. Micrographs were further examined using an image processing software (ImageJ). Density was examined using a helium pycnometer (AccuPyc II 1340).

##### In Vitro Release

Particle dispersions in high-performance liquid chromatography (HPLC)-grade distilled water were prepared at a concentration of 1 mg/mL and kept at 37 °C under 100 rpm shaking using a temperature-controlled shaker. Dispersions were centrifuged at 4000 rpm for 30 min and 1 mL of supernatant was drawn and used to measure the ALD concentration. Spectrophotometric evaluation (CE 7500 Double Beam UV/Visible Spectrophotometer) of the polymeric microspheres was used to calculate the ALD content. The method was based on the formation of an ALD-Cu(II) chromophoric complex absorbing at 240 nm. In solution, alendronate bonds copper ions via chelate bonding. Each alendronate molecule binds to one copper ion, leading to the formation of a chromophore that absorbs photons at the ultraviolet spectrum. This property is exploited for the quantification analysis of alendronate. To account for small amounts of remaining contaminants in the distilled water used, negative control samples were used during the analysis containing empty micro particulate structures of the same composition prepared following the same formulation process without alendronate. These samples were used to form the baseline of the analysis.

##### Biocompatibility Evaluation

Preliminary toxicity experiments were conducted in order to assess the biocompatibility. MC-3T3 cells (ATCC, Teddington, UK) were cultured in culture media; 84.6% v/v Alpha Modified Minimum Essential Medium Eagle (A-MEM), 10% v/v fetal bovine serum (FBS), 2% v/v L-glutamine (200 mM), 2.4% v/v 4-(2-hydroxyethyl)-1-piperazineethanesulfonic acid (1 M), and 1% v/v penicillin/streptomycin solution. Cells were initially grown in polystyrene tissue culture flasks and then transferred to 96-well plates at a concentration of 4 × 10^4^ cells per well to be treated with the polymeric particles. Cells were cultured at 37 °C under 5% CO_2_ atmosphere and all procedures were conducted under aseptic conditions. Particles were sterilized by overnight exposure to ultraviolet radiation.

Metabolic activity evaluation: The impact of the particles on metabolic activity was assessed using the alamarBlue^®^ assay. Briefly, reagent solution was added to the culture at 10% v/v and plates were incubated at 37 °C for 4 h. The fluorescence was measured at an excitation of 540 nm and emission of 620 nm. The results were expressed as a percentage of the value corresponding to untreated cells.

#### 2.2.3. Statistical Analysis

The Thompson Tau analysis was used to determine outliers and the average value and standard deviation of the multiple replicates. Two-tailed unpaired Student’s T-test was performed to establish statistically significant differences; here, *p* ≤ 0.05 are denoted by * and *p* ≤ 0.01 are denoted by **.

## 3. Results

### 3.1. CaP Particles

CaP particles were prepared under different conditions of mixing. The impact of aging on the physical characteristics of the particles was examined. The effects of different homogenization parameters (speed and duration) on the particle size distribution are presented in Table 1.

Under 7000 rpm homogenization speed, an increase in homogenization time leads to a decrease in the surface-weighted average (SWA) and d^50^ of the particle distribution. Longer homogenization times led to a 56% reduction in the SWA diameter of the population. Aging the samples under low stirring under bench conditions leads to a decrease in size at an average of 5.5 μm, regardless of the initial mixing duration. When particles were prepared by mixing at 10,000 rpm, the size distribution remained stable (SWA = 2.7 μm), regardless of the mixing or aging duration.

**Figure 1:**
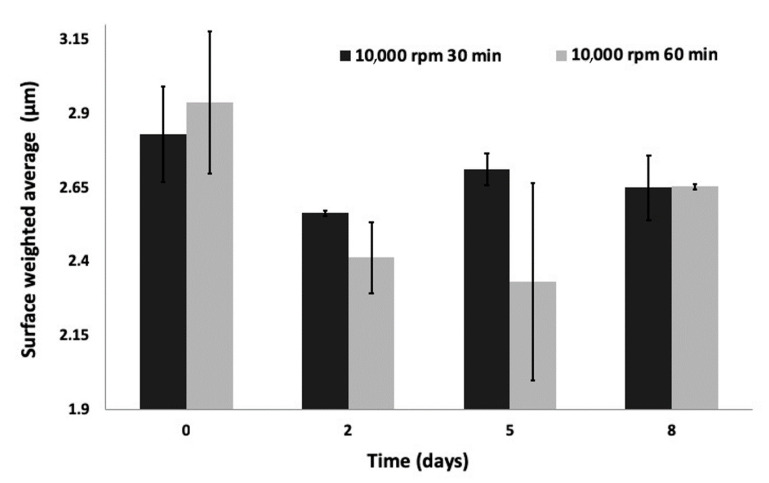
Ca/P particles’ size stability over time under bench conditions. Although statistically significant, the size changes observed due to aging were not significant in magnitude, with the maximum difference observed being 0.6 μm.

The size distribution of samples 3 and 4 were stable for 48 h, and their stability was further examined as presented in Figure 1. Sample 3 parameters were chosen for the continuation of the study. Particle density was evaluated using a helium pycnometer as presented in Table 2.

**Figure 2:**
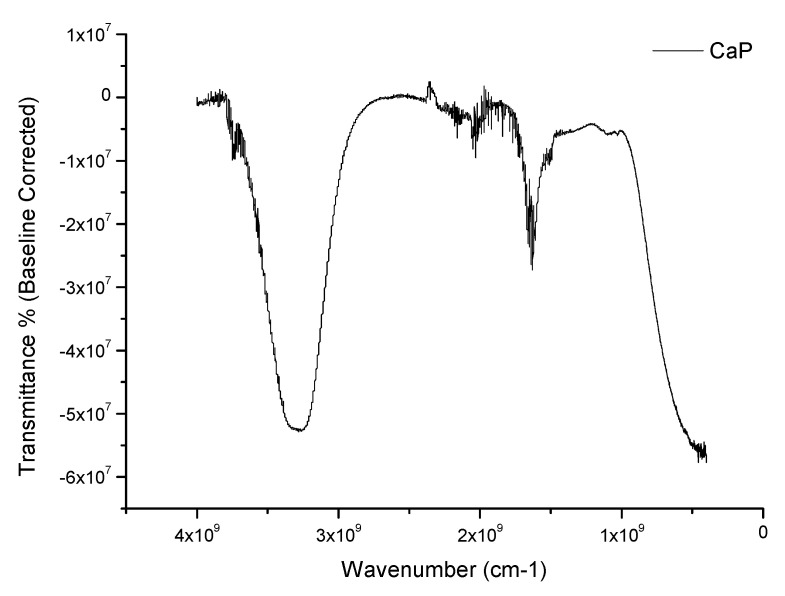
FT-IR spectra of the as-prepared CaP particles prepared following the Mobasherpour protocol. The data underwent baseline correction.

To investigate the identity of the formulated CaP particles, chemical analysis was performed via FT-IR (Figure 2).

**Figure 3:**
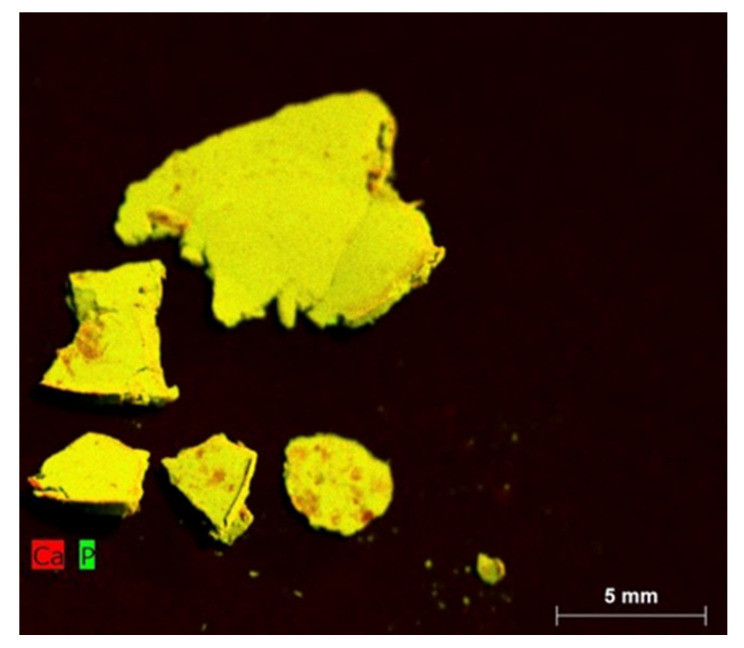
XRF map of the CaP particles as prepared in this study.

By comparing these spectra with the literature, we can identify peaks corresponding to OH^-^ stretching vibration from 3600–2600 cm^−1^, particularly close to 3570 cm^−1^ [10,27,28,29,30], and peaks corresponding to P-OH around 1500 cm^−1^. The wide peak after 3000 cm^−1^ could suggest residual moisture during the analysis as similar peaks appear during dissolution of calcium phosphate [31]. Dry CaP powder was examined by XRD (2θ = 5°–60° at a rate 4°/min using a Cu anode. Although the signal to noise ratio from the acquired diffraction patterns was quite low, two major peaks were observed, around 26° and 31° [10,29]. The same samples were also analyzed using XRF to further investigate the phase of calcium phosphate present (Figure 3).

Although the above analysis exhibited the anticipated calcium to phosphate ratio based on the Mobasherpour protocol [22] followed, further analysis was required to obtain a conclusive identification of these particles in terms of their calcium phosphate phase pattern.

### 3.2. Physical Properties

#### 3.2.1. Physical Characteristics

**Figure 4:**
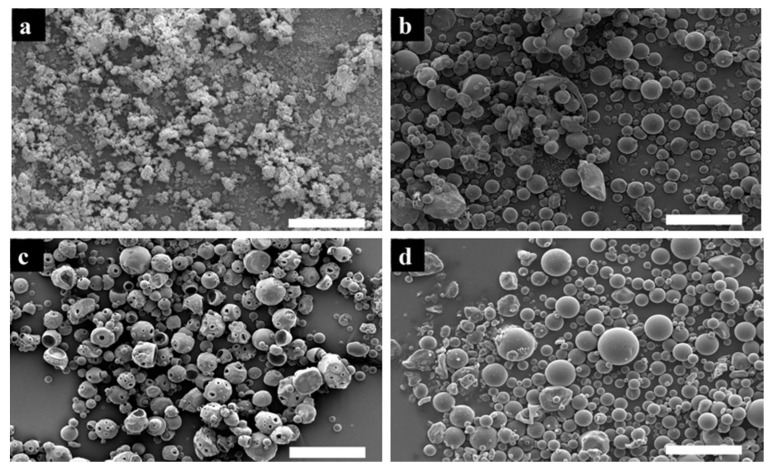
Scanning electron microscopy was used to examine the morphology of particles (**a**) CaP particles, (**b**) poly(lactic-co-glycolic acid) particles (10.525 μm), (**c**) ALD-loaded polymeric particles (10.6 μm), and (**d**) ALD-CaP-loaded PLGA particles (9.13 μm). Scale bar 50 μm.

The encapsulation of ALD, CaP, or their conjugate does not affect the size distribution of the polymeric particles as observed in Figure 4. Encapsulation of ALD appears to have a significant effect on the morphology of the particles; samples of PLGA microspheres demonstrate a small number of porous damaged structures, whilst ALD-loaded microspheres appear to be comprised mostly of highly porous spheres and the incidence of damaged particles increased significantly. The incorporation of ALD in the form of a mineral composite is not accompanied by the same porous structures. In Figure 4d, the structures observed are predominantly spherical with a smooth surface similar to those in Figure 4b.

As porous structures were observed, the density of different compositions was examined using a helium pycnometer. The true density (g/cm^3^) of the samples is presented in Table 2.

The addition of CaP particles within the PLGA polymeric spheres does not have a significant effect on the density of the structures. The encapsulation of ALD decreases the density and thus increases the porosity of the spheres, which is in accordance with the morphological evaluation of their surface (Figure 4c). The encapsulation of ALD in the form of an ALD-CaP conjugate leads to an increase in density. It was also observed that the density of the ALD-CaP–PLGA composites was higher than that of PLGA or CaP-loaded PLGA microspheres.

#### 3.2.2. Encapsulation Efficiency (EE%)

**Figure 5:**
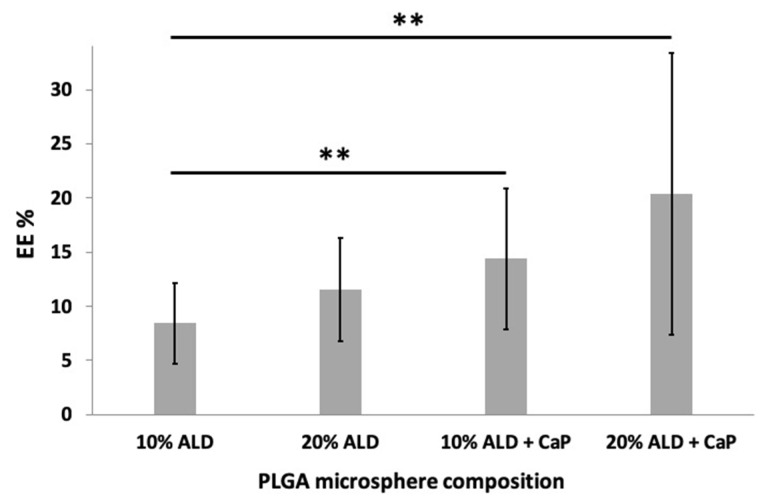
The effect of CaP addition on the encapsulation efficiency (EE%) of ALD within PLGA microspheres under different initial ALD loadings (10% and 20% w/w of PLGA). Six replicates were performed for each composition (** *p* ≤ 0.01).

ALD is highly hydrophilic and consequently not readily retained by polymeric structures, such as PLGA microspheres. As observed in Figure 5, the entrapment efficiency of ALD by polymeric spheres is low regardless of the initial loading. When the initial loading was 10% ALD and 10% ALD CaP, EE% was 8% and 14%, respectively. Then, 20% ALD and 20% ALD CaP gave rise to 11.5% and 20.5% EE%. An increase in loading from 10% to 20% did not have a significant effect on the EE%. Although there is large deviation between samples, the incorporation of ALD in the form of a CaP conjugate leads to a statistically significant increase in the EE%, which is further enhanced when combined with the increase of the initial loading from 10% to 20%.

#### 3.2.3. In Vitro Release

It is important to note that release studies were performed in distilled water instead of a phosphate-buffered saline (PBS), a common buffer solution used in release studies. This decision was based on the fact that the method used for the quantitative analysis of ALD is based on the formation of a chromophoric complex between ALD and copper ions. Cu(II) ions bind to PO_4_^−3^ ions present in PBS, creating a low-solubility chromophoric compound that would interfere with the analysis [32,33]. In the case of CaP-containing structures, this issue is overcome by the fact that the same mass of CaP particles is used for the formation of ALD-loaded samples and empty samples used as blank, thus the effect of the presence of PO_4_^−3^ is subtracted from the final concentration calculations.

**Figure 6:**
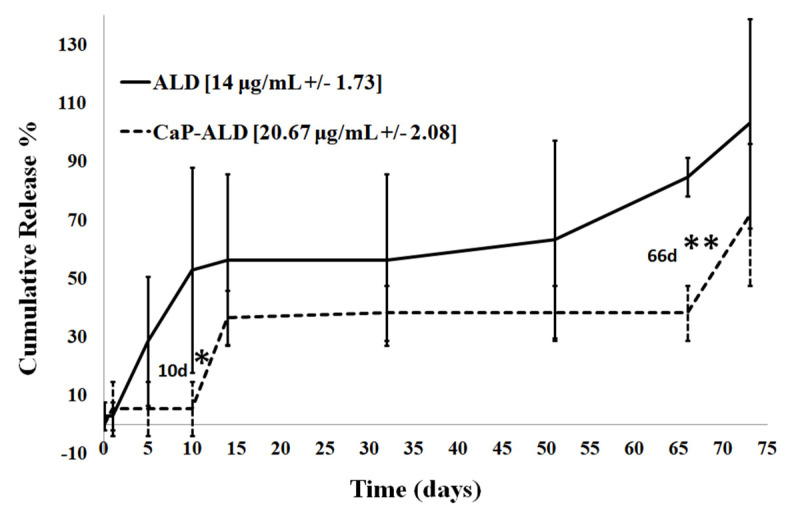
Cumulative release of ALD from PLGA microspheres in vitro. Particles’ suspension was set at a concentration of 1 mg/mL in HPLC-grade distilled water, 37 °C, and suspensions were shaken at a speed of 100 rpm. The final loading of ALD was 14 ug/mL (+/− 1.73) for ALD-loaded PLGA particles and 20.67 μg/mL (+/− 2.08) for ALD-CaP-loaded PLGA particles (* *p* ≤ 0.05 and ** *p* ≤ 0.01).

Figure 6 demonstrates that 50% of ALD is released within the first 10 days from ALD-loaded PLGA microspheres. However, there is little to no burst release from the ALD-CaP-loaded PLGA microspheres. A short burst release was observed in the following five days and the cumulative release remains stable for 50 days. The majority of ALD is released within three months. Statistical analysis showed that there is a statistically significant difference in the release from the two compositions after 10 days and after 2 months.

#### 3.2.4. Cytotoxicity Evaluation

Preliminary toxicological evaluation showed that the introduction of PLGA particles has no negative impact on cellular viability, but on the contrary, there appears to be a positive effect. The values corresponding to treated cells are close or higher than 100% in most cases. Although the increase in the number of particles added per well initially leads to a decrease in cell viability %, after 72 h, the increase of the particulate concentration consistently leads to a statistically significant increase in viability.

Other studies have shown that, although PLGA is generally inert, pre-osteoblasts have not only shown good viability when cultured on PLGA scaffolds but also have exhibited bone-related protein expression and fibril extracellular matrix formation [28]. Protein expression is enhanced by the presence of HA due to its osteoinductive properties. It has also been suggested that the impact of HA could be caused by the roughness of the mineral and increase in the hydrophilicity of systems based on hydrophobic polymers like PLGA. Additionally, a previous co-culture of chondrocytes and osteoblasts in the presence of PLGA and PLGA-bioglass microspheres has shown collagen deposition within the first 10 days with no significant statistical differences between the two systems, proving the positive effect of PLGA on the growth and functionality of bone cells [34]. The viability % increase over 100% in comparison to untreated cells could be an effect of the physical presence of particles increasing the surface area available for cell growth. According to Fu et al., the provision of a biocompatible environment in the vicinity of small cellular clusters can lead to aggregation of the cells in that area and an increase in the proliferation rate [28]. As alamarBlue is a metabolic activity assay, it is limited by the fact that it does not differentiate between an increase in the number of cells or an increase in metabolic activity, which could suggest that the increased signal over the first few days of culture could be because of an increase the in cell number and not due to a change of the metabolic activity. Preliminary toxicological evaluation showed that the introduction of particles has no negative impact on cell viability of pre-osteoblasts.

## 4. Discussion

### 4.1. CaP Particles

In previous studies, it has been demonstrated that CaP particle preparation parameters not only affect the size and morphology of the particles but also the phase of the mineral ranging from amorphous hydroxyl apatite to brushite and finally to highly crystalline HA [22]. Specifically, high-temperature post-formulation treatment leads to an increase in crystallinity and particle size. In order to obtain a significant loading of CaP particles in PLGA microspheres, the size difference between the two structures needs to be maximized. For our formulations, we examined several preparation parameters (mixing speed, mixing duration, and aging duration) in order to obtain the minimum average particle diameter and obtain a narrow size distribution. For these reasons, the preparation was conducted at room temperature and post-formulation heat treatment was avoided to minimize crystal growth.

It was concluded that the size distribution of the particles is affected by preparation parameters; predominately, lower mixing speeds and aging leads to a decrease in size. As agglomerates have been shown to form through cold welding from very fine particles, it was not something that could be easily avoided. In order to decrease the effect that agglomerates have on further usage of the particles, formulations were sonicated prior to utilization and characterization. Although preliminary chemical analysis of those particles was performed using FTIR and XRF analysis, further analysis is required to fully identify the calcium phosphate phases present in these particles. Our study also focused on decreasing the size and crystallinity since highly crystalline HA particles appear to have lower solubility [35], which would hinder the release of the ALD from the CaP particles after they are released from the PLGA microspheres.

The size, morphology, and phase of CaP particles have also been linked to an inflammatory response [36,37]. It has recently been shown that different shapes and sizes of HA particles can influence the recruitment and response of immune cells and signaling molecules. Specifically, smaller particles, less than 10 μm, have been associated with immunological responses; larger particles are less toxic to the surrounding tissue post-injection. The particles formulated in this study were in that range, predominantly to increase incorporation in the PLGA microspheres so this could be an issue in in vivo administration as it can hinder bone remodeling, so it needs to be examined further. Additionally, Lebre’s [37] study also showed that although needle-like fairly sharp particles can have a negative effect on the cell structure as they can damage the cellular membrane, whereas smooth spherical particles do not have the same effect. The particles reported in this research are spherical and therefore should avoid such an immune response.

The incorporation of ALD into PLGA microspheres in the form of CaP conjugates could provide a secondary release mechanism as such particles are soluble in low pH, which makes it an ideal vector for delivering osteoclasts targeting ALD. The local pH in the lacuna created between osteoclast cells and the bone surface is in the range of 4–5 due to the acidic excretions of osteoclasts [35]. Therefore, after the release of CaP particles from PLGA microspheres packed into the fracture site, CaP particles are free to bind to the existing bone surface due to the high affinity of these minerals to bone mineral. The proposed mechanism is as CaP particles dissolve in the lacuna, ALD is gradually released. Once ALD is released into the lacuna, endocytosis is facilitated by the high permeability of the osteoclast’s ruffled membrane. Post-endocytosis, ALD hinders osteoclast activity and consequently the release of ALD from the particles is based on a “feedback” mechanism.

### 4.2. ALD-Loaded PLGA Microspheres

**Figure 7:**
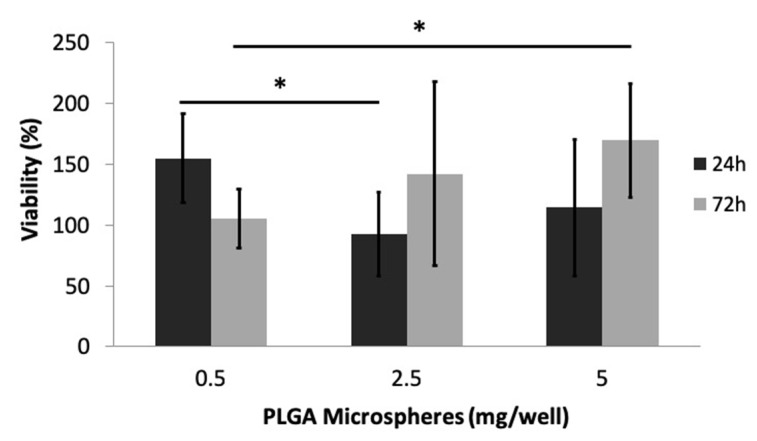
MC-3T3 cellular viability % in the presence of PLGA polymeric microparticles was evaluated over a period of 5 days (* *p* ≤ 0.05).

The chemical composition of the particles significantly affects their physical structure. All compositions led to spherical structures of a similar size distribution, but the surface morphology and the bulk density of the spheres varied. Surface morphology, roughness, and density can all influence the response of a drug delivery system with the host tissue and can also play an important role in the delivery mechanism of the entrapped drug. The incorporation of ALD in the form of a CaP conjugate led to a statistically significant improvement in the encapsulation efficiency (70% increase) of ALD. This is explained by the fact that ALD as a bisphosphonate exhibits a high affinity to bone mineral like HA [38,39]. Additionally, the formulation of less porous structures leads to a decrease in the loss of ALD during fabrication due to diffusion from the inner to the outer aqueous phase. The highly porous structure of ALD-loaded particles explains the initial burst release observed in the first 10 days of the release studies of this system as well as in similar systems [15]. On the contrary, the addition of ALD as a conjugate with CaP particles leads to a significant decrease in porosity, which is used as a way to decrease ALD release via diffusion through the polymeric structure. Most importantly, the study showed that this composite system provides a controlled and prolonged release of ALD that could assist bone healing over a period of 70 days. Finally, preliminary toxicological experiments displayed no significant negative impact on the growth of MC-3T3 cells, a pre-osteoblastic cell line, in the presence of such a structure. The impact of the PLGA degradation in in vitro conditions was tested via the preliminary experiment described in this study (Figure 7). Although, the treated cultures exhibited improved signs of cellular health in comparison to untreated cells and the literature supports the use of these materials for in vivo applications, the authors acknowledge the need for extended experimental studies to evaluate the impact of the developed CaP-PLGA composite structure in cellular and tissue environments to investigate any potential inflammatory-inducing effects before the system can progress to animal studies. Preliminary data suggests that the presence of these structures could promote the growth of these cells, which is explained by the osteoconductive capacity of these materials. The above work supports the safety of these structures in cellular environments.

## 5. Conclusions

This study aimed to develop a composite drug delivery system, which has the ability to deliver ALD to fracture sites in a controlled manner. The system, composed of PLGA ALD-CaP, demonstrated controlled release over 70 days, with increased encapsulation efficiency upon the addition of CaP. Future work will look at developing an injectable carrier system that will allow the delivery of particles to the sites of injury. Extensive studies to determine osteoblast and osteoclast activity, as well as investigating the in vivo effects of the particles on the healing of critical-sized defects, will also be conducted.

## Figures and Tables

**Table 1:** Influence of different homogenization parameters on the Ca/P particle size distribution during time (revolutions per minute (rpm), surface weighted average (SWA)) (*n = 5*).

Samples	Homogenization Parameters		Time (h)	Size Distribution (μm)	
	Speed (rpm)	Duration (min)		SWA	Median Diameter (d^50^)
1	7000	10	-	12.4 +/− 2.274	13.8 +/− 2.354
			48	5.1 +/− 0.349	8.9 +/− 5.322
2	7000	30	-	7 +/− 1.948	9.1 +/− 3.398
			48	6.0 +/− 0.093	7.9 +/− 0.066
3	10,000	30	-	2.8 +/− 0.163	3.1 +/− 0.210
			48	2.6 +/− 0.009	2.8 +/− 0.006
4	10,000	60	-	2.9 +/− 0.239	3.2 +/− 0.407
			48	2.4 +/− 0.332	2.6 +/− 0.057

**Table 2:** Effect of the microparticle chemical composition on the particle density *(n = 5)*.

Sample	Density (g/cm^3^)	
	Mean	SD
CaP	3.14	0.02
PLGA microspheres	1.51	0.13
CaP-loaded PLGA microspheres	1.53	0.01
10% ALD-loaded PLGA microspheres	1.48	0.02
10% ALD-CaP-loaded PLGA microspheres	1.99	0.06

## References

[1] Porter J.R., Ruckh T.T., Popat K.C. (2009). Bone Tissue Engineering: A Review in Bone Biomimetics and Drug Delivery Strategies. Biotechnol. Prog..

[2] Bassi A., Gough J., Zakikhani M., Downes S., Bosworth L.A., Downes S. (2011). Bone tissue regeneration. Electrospinning for Tissue Regeneration.

[3] Klein-Nulend J., Bacabac R.G., Mullender M.G. (2005). Mechanobiology of bone tissue. Pathol. Biol..

[4] Awad H.A., O’Keefe R.J., Lee C.H, Mao J.J., Lanza R., Langer R., Vacanti J. (2014). Bone Tissue Engineering: Clinical Challenges and Emergent Advances in Orthopedic and Craniofacial Surgery. Principles of Tissue Engineering.

[5] Shrivats A.R., Alvarez P., Schutte L., Hollinger J.O., Lanza R., Langer R., Vacanti J. (2014). Bone Regeneration. Principles of Tissue Engineering.

[6] Bose S., Tarafder S. (2012). Calcium phosphate ceramic systems in growth factor and drug delivery for bone tissue engineering: A review. Acta Biomater..

[7] Khan Y., Yaszemski M.J., Mikos A.G., Laurencin C.T. (2008). Tissue Engineering of Bone: Material and Matrix Considerations. J. Bone Jt. Surg. Am..

[8] Bassi A.K., Gough J.E., Zakikhani M., Downes S. (2011). The Chemical and Physical Properties of Poly(ε-caprolactone) Scaffolds Functionalised with Poly(vinyl phosphonic acid-co-acrylic acid). J. Tissue Eng..

[9] Ghag A.K., Gough J.E., Downes S. (2014). The osteoblast and osteoclast responses to phosphonic acid containing poly(ε-caprolactone) electrospun scaffolds. Biomater. Sci..

[10] Chandrasekar A., Sagadevan S., Dakshnamoorthy A. (2013). Synthesis and characterization of nano-hydroxyapatite (n-HAP) using the wet chemical technique. Int. J. Phys. Sci..

[11] Elbackly R.M., Mastrogiacomo M., Cancedda R., Orlando G., Lerut J.P., Soker S., Stratta R.J. (2014). Bone Regeneration and Bioengineering. Regenerative Medicine Applications in Organ Transplantation.

[12] Szpalski C., Sagebin F., Barbaro M., Warren S.M. (2013). The influence of environmental factors on bone tissue engineering. J. Biomed. Mater. Res. B Appl. Biomater..

[13] Silva G.A., Coutinho O.P., Ducheyne P., Reis R.L. (2007). Materials in particulate form for tissue engineering. 2. Applications in bone. J. Tissue Eng. Regen. Med..

[14] Garbuz D.S., Hu Y., Kim W.Y., Duan K., Masri B.A., Oxland T.R., Burt H., Wang R., Duncan C.P. (2008). Enhanced Gap Filling and Osteoconduction Associated with Alendronate-Calcium Phosphate-Coated Porous Tantalum. J. Bone Jt. Surg. Am..

[15] Samdancioglu S., Calis S., Sumnu M., Hincal A. (2006). Formulation and In Vitro Evaluation of Bisphosphonate Loaded Microspheres for Implantation in Osteolysis. Drug Dev. Ind. Pharm..

[16] Shi X., Wang Y., Ren L., Gong Y., Wang D. (2009). Enhancing alendronate release from a novel PLGA/hydroxyapatite microspheric system for bone repairing applications. Pharm. Res..

[17] Kyllönen L., D’Este M., Alini M., Eglin D. (2015). Local drug delivery for enhancing fracture healing in osteoporotic bone. Acta. Biomater..

[18] Messina S., De Simone G., Ascenzi P. (2019). Cysteine-based regulation of redox-sensitive Ras small GTPases. Redox. Biol..

[19] Song S., Cong W., Zhou S., Shi Y., Dai W., Zhang H., Wang X., He B., Zhang Q. (2019). Small GTPases: Stucture, biological function and its interaction with nanoparticles. Asian J. Pharm. Sci..

[20] Coombes A.G.A., Rizzi S.C., Williamson M., Barralet J.E., Downes S., Wallace W.A. (2004). Precipitation casting of polycaprolactone for applications in tissue engineering and drug delivery. Biomaterials.

[21] Nair L.S., Laurencin C.T. (2007). Biodegradable polymers as biomaterials. Prog. Polym. Sci..

[22] Mobasherpour I., Soulati Heshajin M., Kazemzadeh A., Zakeri M. (2007). Synthesis of nanocrystalline hydroxyapatite by using precipitation method. J. Alloy. Compd..

[23] Nasr M., Awad G.A., Mansour S., Shamy A.A., Mortada N.D. (2011). A Reliable Predictive Factorial Model for Entrapment Optimization of a Sodium Bisphosphonate into Biodegradable Microspheres. J. Pharm. Sci..

[24] Mondal T., Sunny M.C., Khastgir D., Varma H.K., Ramesh P. (2012). Poly (l-lactide-co-Є caprolactone) microspheres laden with bioactive glass-ceramic and alendronate sodium as bone regenerative scaffolds. Mater. Sci. Eng. C.

[25] Ostovic D., Stelmach C., Hulshizer B. (1993). Formation of a Chromophoric Complex Between Alendronate and Copper(II) Ions. Pharm. Res..

[26] Perugini P., Genta I., Conti B., Modena T., Pavanetto F. (2001). Long-term Release of Clodronate from Biodegradable Microspheres. AAPS PharmSciTech.

[27] Cheng Z.H., Yasukawa A., Kandori K., Ishikawa T. (1998). FTIR Study on incorporation of CO2 into calcium hydroxyapatite. J. Chem. Soc. Faraday Trans..

[28] Fu B., Sun X., Qian W., Shen Y., Chen R., Hannig M. (2005). Evidence of chemical bonding to hydroxyapatite by phosphoric acid esters. Biomaterials.

[29] Ślósarczyk A., Paszkiewicz Z., Paluszkiewicz C. (2005). FTIR and XRD evaluation of carbonated hydroxyapatite powders synthesized by wet methods. J. Mol. Struct..

[30] Berzina-Cimdina L., Borodajenko N., Theophanides T. (2012). Research of Calcium Phosphates Using Fourier Transform Infrared Spectroscopy. Infrared Spectroscopy–Materials Science, Engineering and Technology.

[31] Boudia S., Zuddas P., Fermane F., Fiallo M., Sharrock P. (2018). Minerological transformation during hydroxyapatite dissolution in simple aqueous solutions. Chem. Biol..

[32] Abou Neel E.A., Ahmed I., Pratten J., Nazhat S.N., Knowles J.C. (2005). Characterisation of antibacterial copper releasing degradable phosphate glass fibres. Biomaterials.

[33] Markich S.J., Brown P.L., Jeffree R.A. (2001). Divalent metal accumulation in freshwater bivalves: An inverse relationship with metal phosphate solubility. Sci. Total Environ..

[34] Jiang J., Tang A., Ateshian G.A., Guo E., Hung C.T, Lu H.H. (2010). Bioactive Stratified Polymer Ceramic-Hydrogel Scaffold for Integrative Osteochondral Repair. Ann. Biomed. Eng..

[35] Matsumoto T., Okazaki M., Inoue M., Yamaguchi S., Kusunose T., Toyonaga T., Hamada Y., Takahashi J. (2004). Hydroxyapatite particles as a controlled release carrier of protein. Biomaterials.

[36] Sabokbar A., Pandey R., Diaz J., Quinn J.M., Murray D.W., Athanasou N.A. (2001). Hydroxyapatite particles are capable of inducing osteoclast formation. J. Mater. Sci. Mater. Med..

[37] Lebre F., Sridharan R., Sawkins M.J., Kelly D.J., O’Brien F.J., Lavelle E.C. (2017). The shape and size of hydroxyapatite particles dictate inflammatory responses following implantation. Sci. Rep..

[38] Sato M., Grasser W., Endo N., Akins R., Simmons H., Thompson D.D., Golub E., Rodan G.A. (1991). Bisphosphonate action. Alendronate localization in rat bone and effects on osteoclast ultrastructure. J. Clin. Investig..

[39] Nancollas G.H., Tang R., Phipps R.J., Henneman Z., Gulde S., Wu W., Mangood A., Russell R.G.G., Ebetino F.H. (2006). Novel insights into actions of bisphosphonates on bone: Differences in interactions with hydroxyapatite. Bone.

